# Repeated exposure to CoCr28Mo6 particles leads to activation of NLRP3 inflammasome signaling in human osteoblasts

**DOI:** 10.1007/s10565-025-10087-7

**Published:** 2025-09-23

**Authors:** Marie-Luise Sellin, Luca Marit Koentopp, Rainer Bader, Anika Jonitz-Heincke

**Affiliations:** Department of Orthopaedics, Biomechanics and Implant Technology Research Laboratory, Rostock University Medical Center, 18057 Rostock, Germany

**Keywords:** Human osteoblasts, Aseptic loosening, Inflammation, NLRP3 inflammasome, Particles

## Abstract

Interleukin (IL)-1β and IL-18 are involved in the inflammatory response of wear-induced osteolysis. The production and secretion of these interleukins are regulated by the nucleotide-binding oligomerization domain leucine-rich repeat-containing protein (NLRP3) inflammasome. Uptake of wear particles can lead to mitochondrial damage, the production of reactive oxygen species (ROS), and stress in the endoplasmic reticulum (ER), resulting in an increased production of the ER stress key factor DNA damage-inducible transcript 3 (DDIT3). This factor is known to inhibit the mitophagy of dysfunctional mitochondria, inducing the generation of ROS. All these factors are known to activate the NLRP3 inflammasome. In this study, we investigated the influence of cobalt-chromium-molybdenum particles (CoCr) on the activation of the NLRP3 inflammasome in human osteoblasts (hOBs). Also, this research aimed to examine the impact of particles on mitochondria and the activation of the inflammasome. HOBs were primed with CoCr particles or tumor necrosis factor (TNF). After the incubation period, the cells were again treated with CoCr particles for activation. To test whether particle-induced DDIT3 upregulation has an effect on mitophagy and regulation of the NLRP3 inflammasome in hOBs, cells were additionally treated with the mitophagy agonist carbonyl cyanide-3-chlorophenylhydrazone (CCCP). Treatment of hOBs with metallic particles increased pyroptosis, which was accompanied by the release of IL-18. Further particle exposure damaged and inhibited the degradation of mitochondria. Activating the mitophagy with CCCP in hOBs reduced the inflammatory response to particles and TNF. These findings indicate that particle-induced inflammation can be influenced by maintaining mitochondrial function.

**Supplementary Information:**

The online version contains supplementary material available at 10.1007/s10565-025-10087-7.

## Introduction

The disruption of bone metabolism, due to inflammatory processes, leads to an imbalance in the activity of osteoblasts and osteoclasts, resulting in a loss of bone mass. During the osteolytic processes associated with aseptic loosening, the differentiation of mesenchymal stem cells (MSCs) into osteoblasts is inhibited (Jiang et al. 2013; Haleem-Smith et al. 2012). Furthermore, the function of osteoblasts is affected by various signals, such as an inflammatory environment and oxidative stress (Yang et al. 2024). Osteoblasts, which can come into contact with wear products at an early stage of aseptic loosening (Xu et al. 2021), reduce bone formation by producing less type 1 collagen and the inactivity of the alkaline phosphatase (ALP) (Lenz et al. 2009; O'Neill et al. 2013). During an inflammatory response, osteoblasts not only contribute to bone loss through increased production of receptor activator of nuclear factor kappa-Β (RANK) ligand (RANKL) (Grimaud et al. 2003), but they can also produce and release inflammatory mediators such as interleukin (IL)−6 and IL-8, thereby exacerbating the inflammatory process (Vallés et al. 2013). It has already been shown that the inflammatory response of macrophages to wear particles is mediated by the nucleotide-binding oligomerization domain leucine-rich repeat-containing protein (NLRP)−3 inflammasome (Burton et al. 2013; Jiang et al. 2021). However, osteoblasts can also form an active NLRP3 inflammasome and release IL-1β and IL-18 (Yang et al. 2024; Brunken et al. 2023; Liu et al. 2020). Inflammasomes are multiprotein complexes composed of proteins of the NOD-like receptor family (NLRs) as sensor proteins, apoptosis-associated speck-like proteins containing a caspase recruitment domain (ASC) as adapter protein, and caspase-1 as effector protein (Wu et al. 2022). The NLRP3 inflammasome is one of the best-studied inflammasomes (Kelley et al. 2019; Chevriaux et al. 2020). The NLRP3 sensor protein consists of three distinct domains: a pyrin domain (PYD), a NACHT domain with ATPase activity, and a leucine-rich repeat domain (LRR). Upon activation of the inflammatory complex, caspase-1 is activated to cleave the preforms of IL-1β, IL-18, and gasdermin D to their mature forms. Gasdermin D is incorporated into the cell membrane as a membrane pore, allowing the release of intracellular mediators (Caicedo et al. 2013; Jiang et al. 2021). Two steps are required to activate the NLRP3 pathway: Firstly, the priming signal that triggers the activation of pattern recognition receptors, such as Toll-like receptors (TLR), leading to the translocation of nuclear factor kB (NF-κB) into the nucleus and initiates the transcription of *NLRP3* and *pro-IL1B* (Kelley et al. 2019). Secondly, a subsequent activation step can be triggered by various stimuli, including adenosine triphosphate (ATP), reactive oxygen species (ROS), or crystalline substances (Caicedo et al. 2013; Jiang et al. 2021; Dostert et al. 2008; Cassel et al. 2008; Hornung et al. 2008; Chen et al. 2023). The activators then initiate the assembly of the NLRP3 inflammasome. In aseptic implant loosening, friction, and corrosion at the bearing surfaces of an artificial joint generate wear particles and metal ions that can induce inflammatory responses (Goldring et al. 1993; Reito et al. 2016). Activation of the inflammasome contributes to the pathogenesis of several inflammatory diseases, including osteolysis and aseptic implant loosening (Mavčič et al. 2020). The uptake of wear debris can lead to the release of several inflammatory mediators, such as IL-1β and IL-18. Wear debris has been implicated as an activator of the inflammasome by inducing the production of ROS or causing lysosomal damage, leading to the release of cathepsin B (Hornung et al. 2008).

It is well known that oxidative stress and ROS trigger inflammatory responses. The release of pro-inflammatory cytokines, such as tumor necrosis factor-alpha (TNF), increases oxidative stress (Huang et al. 2014). In addition, stress in the endoplasmic reticulum (ER) induces the misfolding of proteins (Hetz et al. 2020). One marker of ER stress is DNA damage-inducible transcript 3 (DDIT3), which is produced at low levels in unstressed cells but is elevated in stressed ones (Dong et al. 2024). In osteoblasts, DDIT3 regulates differentiation but can also induce apoptosis during ER stress. Mitochondria produce energy intracellularly by generating ATP through oxidative phosphorylation, which generates ROS. Mitochondrial damage, which can also be caused by wear particles, exacerbates inflammatory processes by leading to mitochondrial calcium overload and excessive ROS production, increasing mitochondrial permeability and inducing pore opening, resulting in the release of damage-associated molecular patterns (DAMPs) (Yin et al. 2024). To remove excess ROS generated by cell stress, damaged or dysfunctional mitochondria are removed by mitophagy. Mitophagy serves to maintain mitochondrial quality and is a mechanism to protect against excessive inflammatory responses (Yin et al. 2024). Upon activation of mitophagy, the PTEN-induced kinase 1 (PINK1)/E3 ubiquitin-protein ligase-parkin (Parkin) signaling pathway is induced, and autophagosome formation is initiated to degrade the damaged components. Dong et al. (2024) were able to establish a link between DDIT3 levels and the initiation of pyroptosis, as DDIT3 inhibits mitophagy in osteoblasts, resulting in the accumulation of large amounts of ROS and triggering the activation of NLRP3 and induction of pyroptosis (Dong et al. 2024).

A previous study (Sellin et al. 2024) showed that priming and activating the NLRP3 inflammasome in human osteoblasts required more than a single stimulus with 0.01 mg/mL particles. However, the study observed that the particles affected NLRP3-associated genes. To demonstrate that wear particles may represent the first and second signal of the inflammasome signaling cascade, this study investigated whether exposure to two doses of CoCr particles triggers activation of the NLRP3 inflammasome. Additionally, the study examined whether a higher dose of particles could enhance inflammasome activation. However, as we could also show that TNF is a strong priming signal in osteoblasts (Sellin et al. 2024), the effects of combined treatment with TNF as a priming signal and a subsequent activation with different CoCr particle concentrations should be further investigated.

Since studies have shown that CoCr particles lead to increased ROS release, an additional focus of our present study is the consideration of mitochondrial markers. We hypothesize that wear particles cause mitochondrial damage and induce ER stress, which may lead to an inhibition of mitophagy. It needs to be clarified whether the associated increase in ROS represents an activation signal for the NLRP3 inflammasome in human osteoblasts.

## Materials & methods

**Fig. 1:**
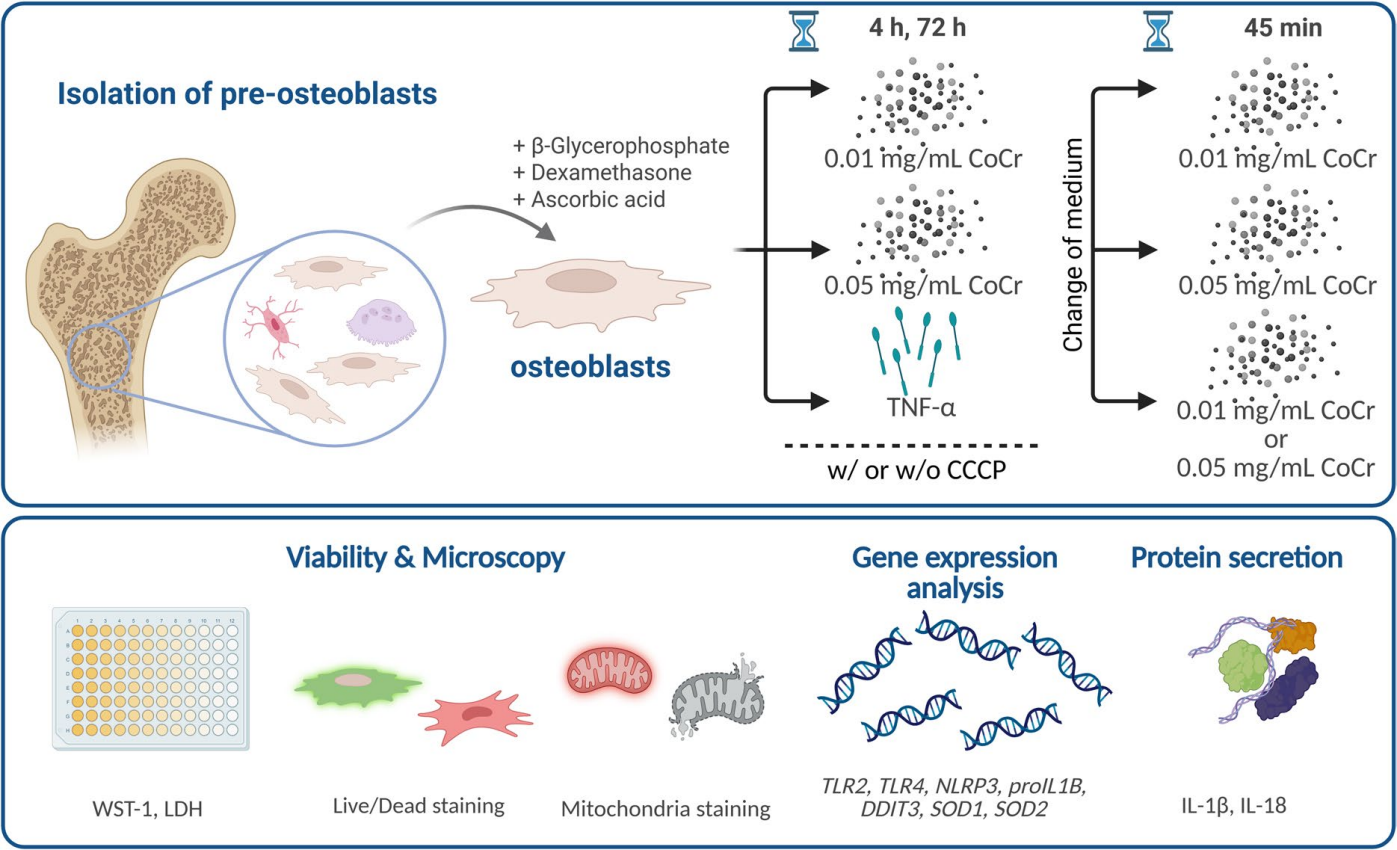
Schematic overview of the experimental setup and the method used. Created in BioRender. Jonitz-Heincke, A. (2025) https://BioRender.com/z64z954

In Fig. 1, an overview of the schematic setup is shown.

## Particle characteristics

In this study, particles (Continuum Blue, Cardiff, UK) derived from a cobalt-chromium-molybdenum alloy (CoCr) were used. According to the manufacturer, the particles were endotoxin-free. To prevent agglomeration, the particles were stored as a 1 mg/mL stock solution in 70% ethanol. Previous studies (Jonitz-Heincke et al. 2018) have analyzed the characteristics of these particles in detail. For the cell culture experiments, the particles were homogenized using ultrasonication (UP100H, Hielscher Ultrasonics GmbH, Teltow, Germany).

## Cell culture of human primary osteoblasts and exposure to metallic particles and TNF

Human osteoblasts were isolated from the femoral heads of patients undergoing total hip arthroplasty according to the established protocol of Lochner et al. (Lochner et al. 2011). Written informed consent was obtained from the patients before the femoral heads were used. The study was approved by the local ethics committee of the University of Rostock (ref. number: A 2010–0010). In total, 36 donors (male: n = 20, age: 67 ± 9 years; female: n = 16, age: 70 ± 7 years) were recruited.

Cells were isolated and cultured under standard culture conditions at 37 °C, 5% CO_2_, and 95% humidity in calcium-free Dulbecco's modified Eagle's medium (DMEM; Pan-biotech, Aidenbach, Germany) supplemented with 10% fetal calf serum (FCS, Pan-biotech, Aidenbach, Germany), 1% amphotericin B, 1% penicillin–streptomycin, and 1% 2-(4-(2-hydroxyethyl)−1-piperazinyl)ethanesulfonic acid (HEPES buffer; all: Sigma-Aldrich, Munich, Germany). To maintain the osteoblastic phenotype, 10 mM β-glycerophosphate, 50 µg/mL ascorbic acid, and 100 nM dexamethasone were added to the cell culture medium before use (all: Sigma-Aldrich, Munich, Germany).

For the cell culture experiments, 30,000 osteoblasts per well of a 24-well plate and 10,000 osteoblasts per well of a 96-well plate were seeded and incubated for 24 h.

After 24 h of attachment, osteoblasts were treated with 0.01 mg/mL cobalt-chromium-molybdenum (CoCr) particles, 0.05 mg/mL CoCr particles, or 50 ng/mL TNF (abcam, Cambridge, UK). The particle concentrations were selected based on previous studies (Klinder et al. 2018; Sellin et al. 2024; Jonitz-Heincke et al. 2018). The particles had an average size of 0.5 µm and were irregularly shaped with a flake-like to globular appearance (Klinder et al. 2018). Osteoblasts in cell culture medium were used as a negative control. Cells were pretreated for 4 or 72 h and then activated for 45 min. The time points for priming and activation were chosen based on a previous study (Sellin et al. 2024) and were optimized in our laboratory. The stimulation medium was removed and replaced with a medium containing a further 0.01 mg/mL or 0.05 mg/mL of CoCr particles.

The effect on mitophagy was investigated using the mitophagy agonist carbonyl cyanide 3-chlorophenylhydrazone (CCCP, 5 µM, ThermoFisher Scientific, Waltham, MA, USA). Cells were incubated directly with TNF or CoCr (0.01 mg/mL) together with CCCP for 72 h.

## Determination of metabolic activity

Cell viability was determined using a water-soluble tetrazolium salt (WST-1; TakaraBio, London, UK). After the incubation period, the supernatant was removed, and the cells were diluted with phosphate-buffered saline (PBS; Biochrom AG, Berlin, Germany) and with WST-1 reagent at a 1:10 dilution with cell culture medium for 45 min. Then, 100 µL of the supernatant was transferred in duplicate to a 96-well plate, and the absorbance was measured at 450 nm (reference: 630 nm) in a microplate reader (Tecan Group AG, Männedorf, Switzerland).

## LDH assay

Since lactate dehydrogenase (LDH) activity serves as a marker of inflammasome activity and pyroptosis induction, an LDH activity assay (Sigma-Aldrich, Munich, Germany) was performed. To perform the assay, 50 µL of the supernatant was transferred to a new 96-well plate, and the assay was performed according to the manufacturer's instructions. Absorbance was measured at a wavelength of 450 nm using a Tecan Infinite® 200 Pro microplate reader (Tecan Group AG, Maennedorf, Switzerland).

## Live-Dead staining

Live-dead staining (Invitrogen, Carlsbad, CA, USA) was performed to differentiate and quantify living and dead cells. After the incubation period, the medium was removed, and the cells were washed with PBS. The cells were then incubated with PBS containing calcein (1:4000) and ethidium Homodimer 1 (EthD-1; 1:1000) for 30 min in the dark. Live staining was then measured at 495 nm and dead staining at 630 nm in a plate reader (Tecan Group AG, Männedorf, Switzerland). In addition, the cells were imaged with a Keyence BZ-X810 microscope (Keyence Germany GmbH, Neu-Isenburg, Germany) with a 20 × objective.

## Staining of mitochondria

The dye tetramethylrhodamine (TMRM) (Invitrogen, Carlsbad, CA, USA) was used to stain active mitochondria. After the treatment times, osteoblast medium containing the dye was added to the cells at a ratio of 1:1000. The cells were incubated for 30 min at 37 °C, washed, and then incubated for 10 min with Hoechst 33342 (Immunochemistry Technologies, Davis, CA, USA) to stain the nuclei of osteoblasts. For quantitative evaluation, mitochondrial staining was measured at a wavelength of 630 nm on a plate reader (Tecan Group AG, Maennedorf, Switzerland). Microscopic evaluation was performed using a Keyence BZ-X810 microscope (Keyence Germany GmbH, Neu-Isenburg, Germany) with a 20 × or 40 × objective.

## Actin-DAPI staining

To analyze morphological changes of the human osteoblasts, actin staining with diamidino-2-phenylindole dihydrochloride (DAPI) counterstain was used to visualize the structure of the cytoskeleton after exposure to particles and TNF. After removing the medium and washing with PBS (biochrom, Berlin, Germany), the cells were fixed with 4% paraformaldehyde (PFA; pH: 7.0) for 10 min and further rinsed with PBS for 30 s. Subsequently, the cell membrane was permeabilized by adding a permeabilization buffer containing 0.05% Triton-X (Merck KGaA, Darmstadt, Germany) for 5 min. Before adding the 100 nM actin staining solution (100 nM Acti-Stain 488 Fluorescent Phalloidin, Cytoskeleton, Denver, CO, USA) for 30 min, the cells were rewashed three times with PBS. The counterstain of the nuclei was done with DAPI (Merck KGaA, Darmstadt, Germany) for 5 min. The staining solution was removed, and the cells were washed and stored at 4 °C.

The microscopic examination was performed using the CytoViva® Enhanced Darkfield Hyperspectral Microscope System (CytoViva, Inc., Auburn, AL, USA) and a 60 × oil objective. With CytoViva’s dual-mode fluorescence module, simultaneous real-time observation of fluorescent and non-fluorescent sample components is possible. The bandpass emission filter (69002m, Chroma Technology Corporation, Bellow Falls, VT, USA) allows imaging of the cytoskeleton of the cells at a wavelength of 525 nm and the nuclei at a wavelength of 461 nm. The darkfield modus was used to visualize unstained structures such as intracellular vesicles, large endosomes, and granules. For image acquisition, the Ocular Imaging software (Teledyne Photometrics, Tucson, AZ, USA) was used.

## Staining of NLRP3

To investigate NLRP3 expression, osteoblasts were fixed with 4% PFA for 10 min after treatment. Then, the cells were blocked and permeabilized with 0.3% Triton X-100 (Merck KGaA, Darmstadt, Germany) and 5% goat serum (abcam, Cambridge, UK). After an hour-long incubation period, the cells were washed three times for five minutes each with PBS. Next, NLRP3 was stained by adding an NLRP3 antibody (ab283819, abcam, Cambridge, UK) diluted 1:50 with 1% goat serum. The cells were incubated overnight at 4°C. In the dark, the primary antibody was removed, and the cells were washed three times with PBS. Then, the secondary antibody, Alexa Fluor 594 goat anti-rabbit (ab150080, abcam, Cambridge, UK), was added at a dilution of 1:500 with 1% goat serum. After two hours, the solution was removed, and the cell nuclei were stained with DAPI, as described above. Slides were examined using a CytoViva® Enhanced Darkfield Hyperspectral Microscope System (CytoViva, Inc., Auburn, AL, USA) with a 60 × oil objective.

## Gene expression analysis

Total RNA was isolated using the innuPREP RNA Mini Kit 2.0 (Analytik Jena GmbH, Jena, Germany) according to the manufacturer's protocol. The isolated RNA concentration was determined using the Tecan Infinite® 200 Pro microplate reader and NanoQuant Plate™ (both: Tecan Group AG, Männedorf, Switzerland). RNase-free water was used as a blank. Subsequently, 100 ng of RNA from each sample was transcribed into complementary deoxyribonucleic acid (cDNA) using the High Capacity cDNA Reverse Transcription Kit (Applied Biosystems, Forster City, CA, USA). The following PCR protocol was used: 10 min at 25 °C, 120 min at 37 °C, and 5 min at 85 °C in a thermocycler (Analytik Jena GmbH, Jena, Germany). The samples were then diluted with 20 µL RNase-free water and stored at −20°C.

Gene expression levels were determined by semiquantitative real-time polymerase chain reaction (qPCR, qTower 2.0, Analytik Jena GmbH, Jena, Germany) using innuMIX qPCR Masterix SyGreen (Analytik Jena AG, Jena, Germany) and the following primer pairs: NLRP3 (NM_001079821.3) fwd: 5'-AGGAGAACTTTCTGTGTGGACC-3'; rev: 5'-TTCTCTGTCTGACCCCTCGG-3'; pro-IL1B (NM_000576.3) fwd: 5'- TACTCACTTAAAGCCCGCCT-3'; rev: 5'- ATGTGGGAGCGAATGACAGA-3'; proIL-18 (NM_001243211.2) fwd: 5`- TGCAGTCTACACAGCTTCGG-3'; rev: 5'- GCAGCCATCTTTATTCCTGCG-3'; β-Actin (NM_001101.5) fwd: 5'- CTTCCTGGGCATGGAGTC −3'; rev: 5'- AGCACTGTGTTGGCGTACAG-3'; TLR2 (NM_001318787.2) fwd: 5'-GGAGTTCTCCCAGTGTTTGGT-3'; rev: 5'-TTCCTGCCTTCACTTGGTCA-3'; TLR4 (NM_003266.4) fwd: 5'-CCCTTCACCCCGATTCCATT-3'; rev: 5'-TTGTCTGGATTTCACACCTGGA-3'; DDIT3 (NM_001195053.1) fwd: 5'- ACCTGAGGAGAGAGTGTTCAAG-3'; rev: 5'-GCAGGATAATGGGGAGTGGC-3'; SOD2 (NM_000636.4) fwd: 5'- GCTGGAAGCCATCAAACGTG-3'; rev: 5'-GCAGTGGAATAAGGCCTGTTG-3'.

Each sample was measured in duplicates. Ultrapure water served as the negative control. Each primer pair was tested for duplicates. The following qPCR protocol was used: 95 °C for 2 min, followed by 40 cycles of denaturation for 5 s at 95 °C and annealing/extension for 25 s at 60–65°C. The relative amount of mRNA compared to β-actin as housekeeping gene was calculated using the equation ΔCt = Ct_target_-Ct_housekeeping gene_. The relative expression of the gene in the treated samples was calculated using the 2^−ΔΔCt^ method (relative to the untreated control).

## Quantification of secreted proteins

After the respective treatment times, the supernatants were collected to determine the released concentration of IL-1β (Human IL-1-beta ELISA Ready-SET-Go!™, ThermoFisher Scientific, Waltham, MA, USA) and IL-18 (Human IL-18 Antibody Pair Kit, Invitrogen, Carlsbad, CA, USA). The assays were performed according to the manufacturer's instructions. The absorbance was measured at 405 nm using a Tecan Infinite® 200 Pro microplate reader (Tecan Group AG, Maennedorf, Switzerland). The concentration in the samples was calculated from a standard curve. Normalization of protein content to total protein was performed using the Qubit Protein Assay Kit and Qubit 1.0 (both Thermo Fisher Scientific, Waltham, MA, USA) according to the manufacturer's protocol.

## Graphical illustration and statistics

The experiments were performed with primary osteoblasts from 36 individual donors. Unless otherwise described, data were presented as individual data (one data point per donor) with median and interquartile ranges. Statistical analysis was performed with GraphPad Prism, version 9.0 (GraphPad Software, San Diego, CA, USA). If not otherwise stated, the different treatments and periods were compared with each other using two-way ANOVA with Bonferroni's multiple comparison post Hoc test. A p-value of less than 0.05 was defined as statistically significant.

## Results

### Influence of TNF and CoCr particles on the viability of human osteoblasts

**Fig. 2:**
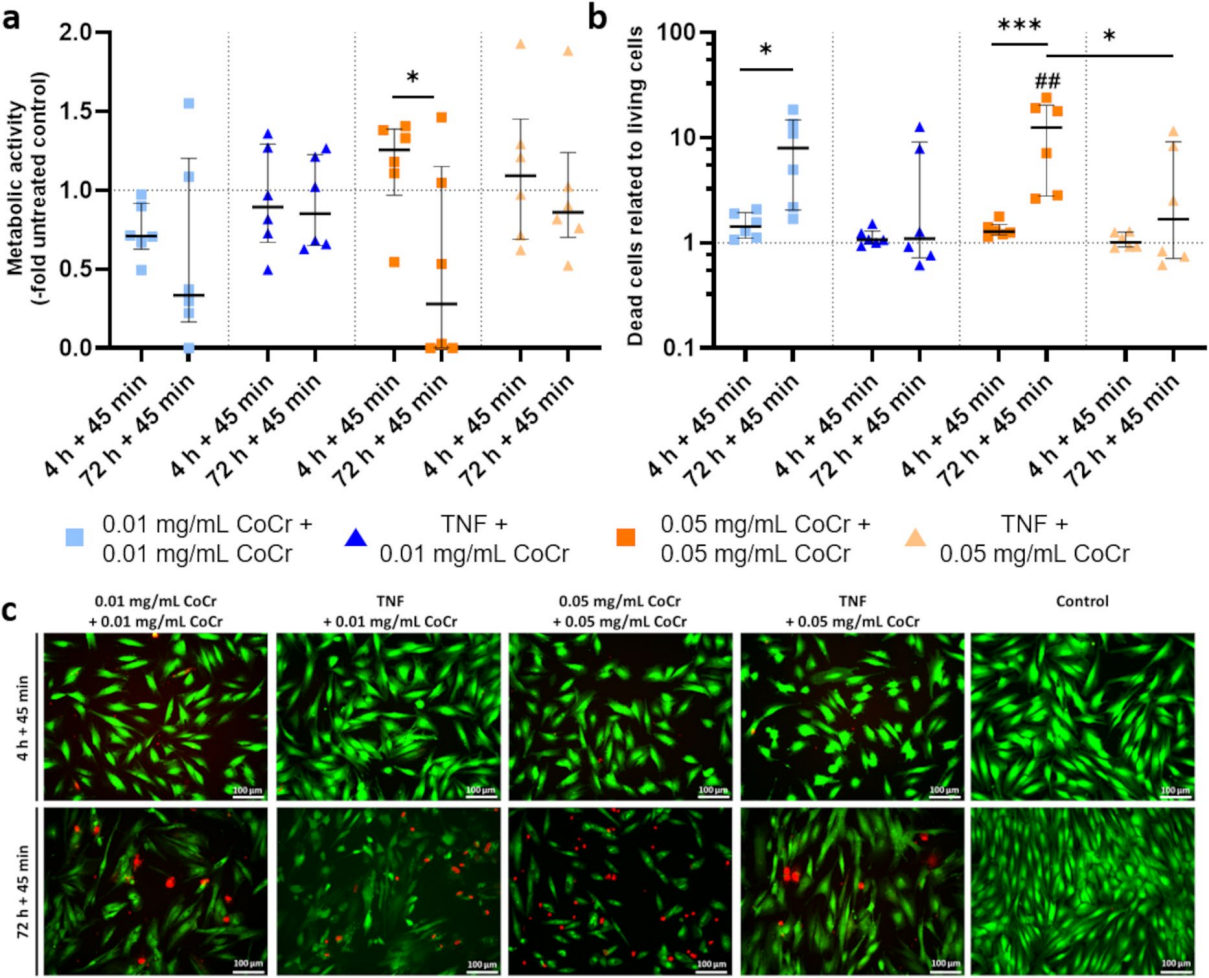
Quantification of metabolic activity **a** and the ratio of dead to live cells **b** with representative microscopic evaluation **c** after treatment of human osteoblasts. Osteoblasts (n = 6) were treated with TNF or CoCr particles for 4 h or 72 h and then activated with CoCr particles for 45 min. Untreated cells served as controls. **a** Metabolic activity was determined by WST-1 assay. **b**, **c** The percentage of live (green) and dead (red) cells was determined by calcein-ethidium Homodimer 1 staining. The microscopic pictures were taken with the Keyence BZ-X810 microscope with a 20 × objective. Two-way ANOVA determined statistical significance with Bonferroni post hoc test: ****p < 0.0001, **p < 0.01, *p < 0.05 (Significance between the time points); ^####^p < 0.0001, ^##^p < 0.01 (Significance to untreated control); Bar: 100 µm

Treatment of osteoblasts with 0.01 mg/mL CoCr particles resulted in reduced metabolic activity compared to the untreated control. Pretreatment with TNF followed by activation with 0.01 mg/mL or 0.05 mg/mL particles did not significantly change the metabolic activity. Double stimulation of osteoblasts with 0.05 mg/mL for 4 h + 45 min resulted in a slight increase in activity. In comparison, the metabolic activity was significantly reduced after 72 h + 45 min (p = 0.0212)(Fig. 2a). Live-dead staining (Fig. 2b) revealed a significant increase in the proportion of dead cells after treatment with 0.01 mg/mL CoCr and 0.05 mg/mL for 72 h + 45 min compared to untreated cells (p_0.05mg/mL_ = 0.0026) and compared to 4 h + 45 min treatment (p_0.01mg/mL_ = 0.0370; p_0.05 mg/mL_ = 0.0009). At 72 h + 45 min, significantly more dead cells were detected after double 0.05 mg/mL CoCr treatment compared to TNF + 0.05 mg/mL treatment (p = 0.0248). Microscopic images confirmed the measurements (Fig. 2c).

### Influence on the expression of TLR and NLRP3 inflammasome-associated genes

**Fig. 3:**
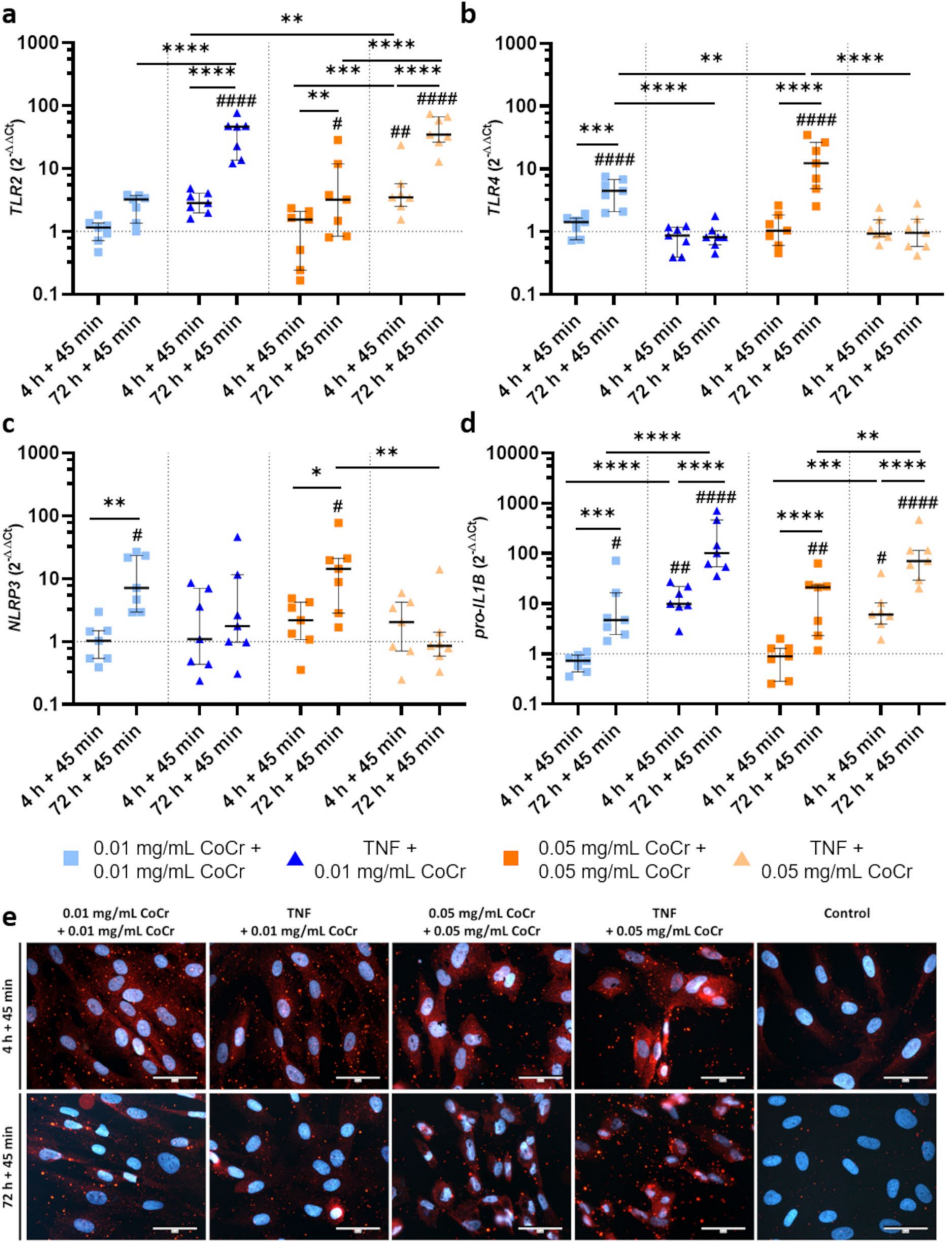
Analysis of NLRP3 inflammasome-associated genes **a**-**d** and expression of NLRP3 **e** after exposure of human osteoblasts to TNF or CoCr particles for 4 h or 72 h, followed by retreatment with CoCr particles for 45 min. NLRP3 expression was identified by staining NLRP3 and counterstaining the nuclei with DAPI. The representative microscopic pictures were taken with the CytoViva® Enhanced Darkfield Hyperspectral Microscope System with a 60 × objective. **a**-**d** Data (n = 7) were calculated using the 2^−ΔΔCt^ method (-fold untreated control, dashed line). Statistical significance was determined by two-way ANOVA followed by Bonferroni post hoc test: ****p < 0.0001, ***p < 0.001, **p < 0.01, *p < 0.05; ^####^p < 0.0001, ^##^p < 0.01, ^#^p < 0.05 (Significance to untreated control). Bar: 50 µm

Osteoblasts treated with TNF + 0.01 mg/mL CoCr particles expressed significantly more *TLR2* mRNA (Fig. 3a) after 72 h + 45 min compared to 4 h + 45 min (p < 0.0001) and untreated cells (p < 0.0001), as well as compared to the double treatment with 0.01 mg/mL CoCr (p < 0.0001). Double treatment with 0.05 mg/mL resulted in a significant increase in *TLR2* expression compared to 4 h + 45 min treatment (p = 0.0035) and untreated cells (p = 0.0328). At the same time, the double stimulation with 0.05 mg/mL resulted in a significantly lower *TLR2* expression compared to TNF + 0.05 mg/mL (p < 0.0001). After 4 h + 45 min, the gene expression of *TLR2* was significantly increased after TNF + 0.05 mg/mL CoCr compared to TNF + 0.01 mg/mL CoCr (p = 0.0050), 0.05 mg/mL CoCr + 0.05 mg/mL CoCr (p = 0.0008), and to the untreated control (p < 0.0001). Further, treatment of cells with TNF + 0.05 mg/mL CoCr resulted in a significant increase in mRNA levels at 72 h + 45 min compared to 4 h + 45 min treatment (p < 0.0001), the double treatment with 0.05 mg/mL particles (p < 0.0001), and untreated control (p < 0.0001).

Compared to untreated cells (p < 0.0001), 4 h + 45 min (p = 0.0003) and TNF + 0.01 mg/mL CoCr (p < 0.0001), *TLR4* expression was significantly increased after 72 h + 45 min with 0.01 mg/mL CoCr (Fig. 3b). A concentration-dependent increase in *TLR4* levels was detected, as mRNA levels were significantly higher after 72 h + 45 min with double 0.05 mg/mL CoCr treatment compared to double 0.01 mg/mL treatment (p = 0.0097). Furthermore, the expression was significantly increased after 72 h + 45 min 0.05 mg/mL CoCr + 0.05 mg/mL CoCr compared to 4 h + 45 min (p < 0.0001), untreated control (p < 0.0001), and TNF + 0.05 mg/mL (p < 0.0001).

While 72 h + 45 min treatment of cells with particles resulted in significantly increased mRNA levels of *NLRP3* compared to untreated control (0.01 mg/mL: p = 0.0365; 0.05 mg/mL: p = 0.0143) and 4 h + 45 min stimulation (0.01 mg/mL p = 0.0032; 0.05 mg/mL p = 0.0265), treatment with TNF + CoCr did not alter expression significantly (Fig. 3c). Treatment of cells with TNF + 0.05 mg/mL CoCr resulted in significantly decreased gene expression compared to 72 h + 45 min 0.05 mg/mL CoCr treatment (p = 0.0046). To verify whether human osteoblasts synthesize NLRP3, the protein was stained in the cells (Fig. 3e). Microscopic images revealed stronger staining of the cells after treatment compared to the untreated control. While the osteoblasts showed a positive signal after 4 h + 45 min in all stimulation groups, positive staining was particularly evident after 72 h + 45 min in the samples treated with 0.05 mg/mL particles.

The expression of *pro-IL1B* (Fig. 3d) showed a time-dependent increase at 72 h + 45 min for all treatments. Exposure of osteoblasts to 0.01 mg/mL particles for 4 h + 45 min resulted in significantly decreased expression of *pro-IL1B* transcripts compared to 72 h + 45 min (p = 0.0003). After 72 h + 45 min, osteoblasts treated twice with 0.01 mg/mL CoCr particles showed significantly increased mRNA levels compared to untreated cells (p = 0.0327). Pretreatment with TNF and subsequent activation with 0.01 mg/mL CoCr particles resulted in significantly increased *pro-IL1B* expression rates compared to the untreated control (4 h + 45 min p = 0.0019; 72 h + 45 min p < 0.0001) and double treatment with 0.01 mg/mL particles (all: p < 0.0001). Furthermore, a time-dependent effect (p < 0.0001) was observed in this stimulation group. Dual treatment of osteoblasts with 0.05 mg/mL CoCr particles resulted in significantly lower mRNA expression of *pro-IL1B* at 4 h + 45 min compared to 72 h + 45 min (p < 0.0001) and decreased expression compared to TNF + 0.05 mg/mL CoCr (4 h + 45 min: p = 0.0008; 72 h + 45 min: p = 0.0019). After exposure of osteoblasts to double 0.05 mg/mL CoCr (72 h + 45 min: p = 0.0039) and TNF + 0.05 mg/mL CoCr (4 h + 45 min: p = 0.0224; 72 h + 45 min: p < 0.0001) exposure, significantly increased expression levels were present compared to the untreated control. In addition, a time-dependent difference was observed after TNF + 0.05 mg/mL (p < 0.0001).

### Activity of LDH and secretion of IL-18 after exposure to TNF and CoCr particles

**Fig. 4:**
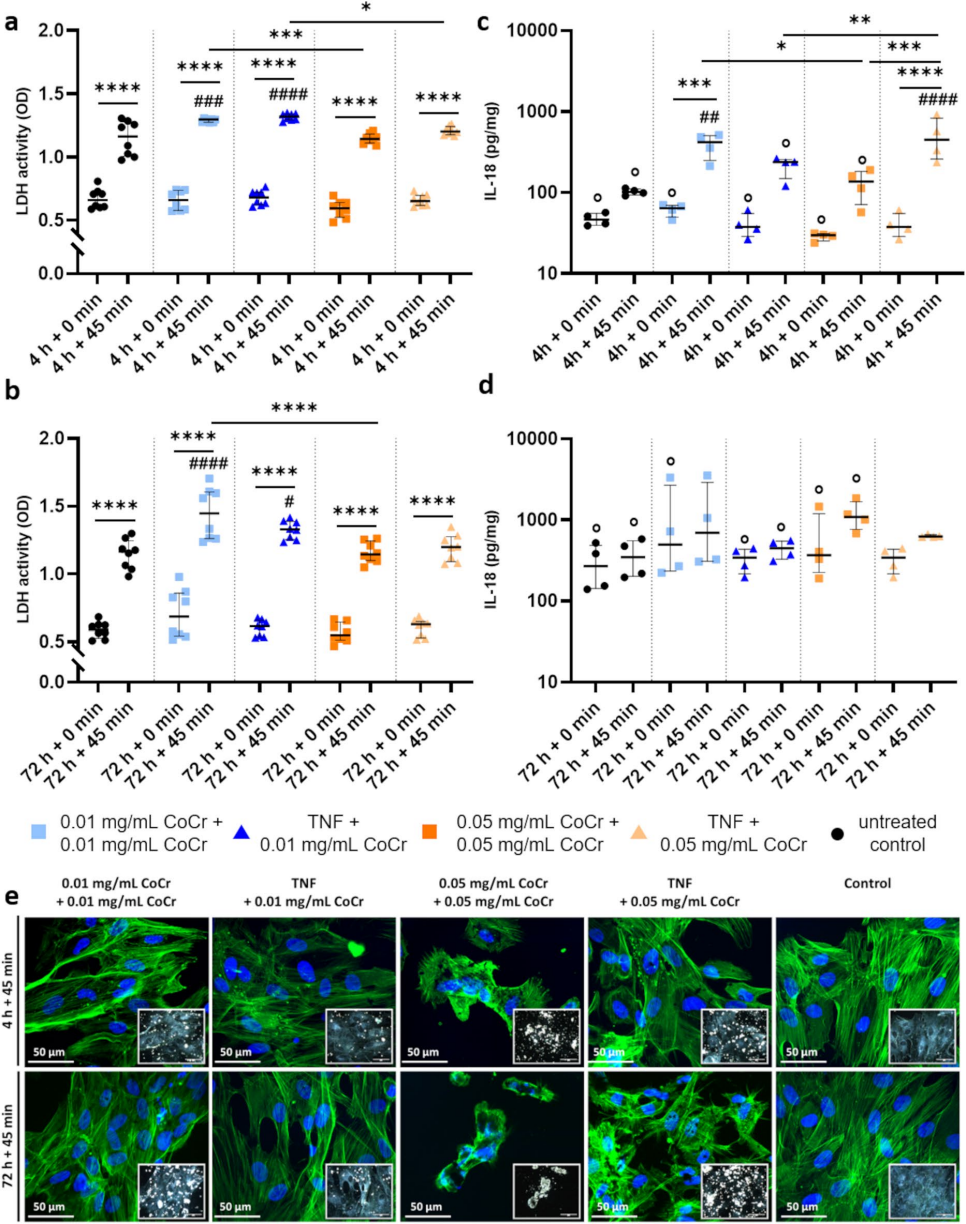
Determination of LDH activity **a** and the release of IL-18 **b**-**c**, as well as microscopic images **d** after treatment of human osteoblasts (n ≥ 4) with TNF or CoCr particles and subsequent activation with CoCr particles. Morphological changes were identified by staining the cytoskeleton with Phalloidin and counterstaining the nuclei with DAPI. The picture in the lower right corner shows a darkfield image of the osteoblasts exposed to the different stimuli. The bright, white specks indicate the location of the particles. The microscopic pictures were taken with the CytoViva® Enhanced Darkfield Hyperspectral Microscope System with a 60 × objective **d**. Statistical significance was determined by two-way ANOVA followed by Bonferroni as a post hoc test **a**-**d**. For comparisons between 4 h ± 45 min and 72 h ± 45 min, the Shapiro–Wilk test was first used to test for normal distribution and then the Mann–Whitney test was used to determine statistical significance **c**-**d**: ***p < 0.001, *p < 0.05; ^####^p < 0.0001 ^##^p < 0.01 (Significance to untreated control of the corresponding time point); °p < 0.05 (Significance between 4 and 72 h or 4 h + 45 min and 72 h + 45 min between c and d); Bar: 50 µm

LDH activity (Fig. 4a, b) was significantly lower after 4 h compared to 4 h + 45 min in all treatment groups (all: p > 0.0001). In addition, LDH activity was significantly increased after 4 h + 45 min with the lower particle concentration compared to the untreated control (p = 0.0006) and 0.05 mg/mL + 0.05 mg/mL particles (p = 0.0005) (Fig. 4a). Furthermore, pretreating the cells with TNF, followed by activation with 0.01 mg/mL particles, resulted in significantly higher activity compared to the untreated control (p > 0.0001) and the activation with 0.05 mg/mL particles (p = 0.0166).

All treatments showed significantly increased LDH activity after an additional 45-min treatment (all: p < 0.0001) (Fig. 4b). After exposing osteoblasts to 0.01 mg/mL CoCr + 0.01 mg/mL CoCr, a significant increase in LDH activity was observed after 72 h + 45 min, compared to the untreated control and 0.05 mg/mL CoCr + 0.05 mg/mL CoCr (all: p < 0.0001). Additionally, increased LDH activity was detected after 72 h + 45 min following TNF at 0.01 mg/mL CoCr compared to the untreated control (p = 0.0149).

The release of IL-18 (Fig. 4c, d) was examined before and after the re-addition of CoCr particles for 45 min. A time-dependent effect was observed, as after 72 h (Fig. 4d), a significantly increased concentration of IL-18 was measured in the untreated controls, single treatment with 0.01 mg/mL CoCr, TNF ± 0.01 mg/mL CoCr and 0.05 mg/mL CoCr ± 0.05 mg/mL CoCr treatment (all: p = 0.0286) compared to the four-hour treatment (Fig. 4c). Osteoblasts stimulated with 0.01 mg/mL CoCr particles for 4 h + 45 min showed significantly increased IL-18 release compared to single treatment after 4 h (p = 0.0008), to 0.05 mg/mL CoCr + 0.05 mg/mL CoCr (p = 0.01932), and the untreated control (0.0073). In addition, there was an increased release following TNF + 0.05 mg/mL CoCr exposure after 4 h + 45 min compared to single TNF treatment (p < 0.0001), TNF + 0.01 mg/mL CoCr (p = 0.0054), and double 0.05 mg/mL CoCr treatment (p = 0.0003).

Staining of the actin cytoskeleton revealed morphological changes (Fig. 4e). Intact osteoblasts of the untreated control showed a polygonal, elongated shape with long, regular actin fibers. Changes in cell morphology were particularly evident in osteoblasts exposed to 0.05 mg/mL CoCr. After double particle treatment, the cells had an irregular shape with short and irregular actin fibers as early as 4 h. In addition, the cells were smaller and had short cytoplasmic extensions. The effect increased with time, and a loss of cellular organization was observed after 72 h. Osteoblasts incubated twice with 0.01 mg/mL CoCr showed thickened actin filaments.

### Influence of treatments on mitochondrial genes and the mitochondrial membrane potential

**Fig. 5:**
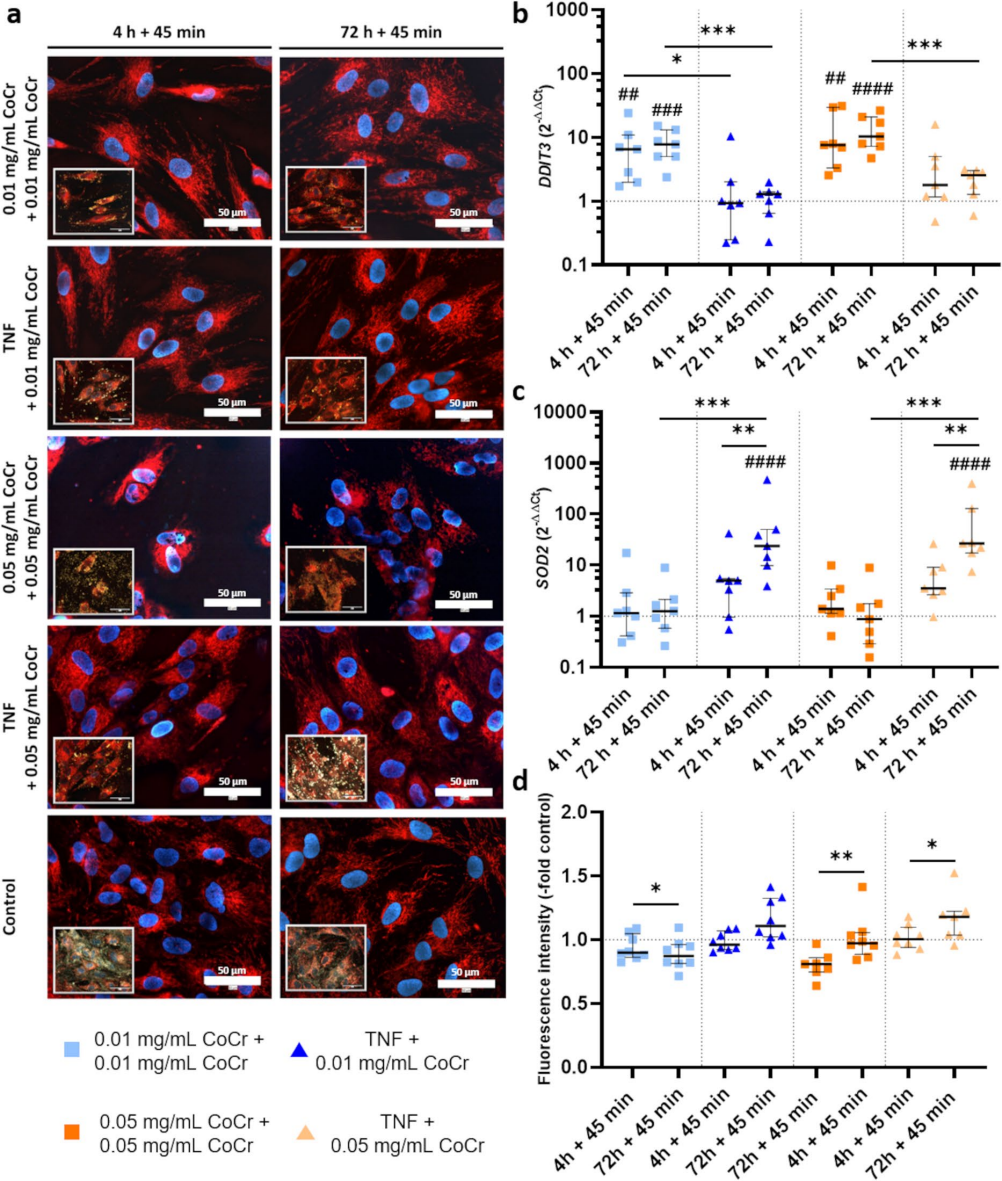
Determination of gene expression of *DDIT3* **b** and *SOD2* **c** as well as mitochondrial membrane potential **a**; **d** after priming of human osteoblasts (n ≥ 7) with CoCr particles or TNF and subsequent activation with CoCr particles. Microscopic pictures were taken with the CytoViva® Enhanced Darkfield Hyperspectral Microscope System with a 60 × objective. In the picture in the lower corner, the fluorescence image is combined with the darkfield image to visualize the location of the particles. The particles appeared as bright white spots. **b**, **c** Gene expression data were calculated using the 2^−ΔΔCt^-method (-fold untreated control, dashed line). **d** The effects of exposure on mitochondrial membrane potential were analyzed by TMRM staining. Statistical significance was determined by two-way ANOVA followed by Bonferroni post hoc test: ***p < 0.001, **p < 0.01, *p < 0.05; ^####^p < 0.0001, ^###^p < 0.001, ^##^p < 0.01 (Significance to untreated control). Bar: 50 µm

The function of mitochondria after treatment with CoCr particles and TNF was examined. Next to the analysis of the mitochondrial membrane potential and mitophagy, gene expression profiles of superoxide dismutase 2 (*SOD2*) and DNA damage-inducible transcript 3 (*DDIT3*) were obtained (Fig. 5).

The gene expression of *DDIT3* was significantly increased after particle treatment compared to untreated cells after 4 h + 45 min (0.01 mg/mL: p = 0.0046; 0.05 mg/mL: p = 0.0009) and 72 h + 45 min (0.01 mg/mL: p = 0.0006; 0.05 mg/mL: p < 0.0001) (Fig. 5b). Particle exposure caused a significant upregulation of the gene expression after 4 h + 45 min (0.01 mg/mL: 0.0044) and 72 h + 45 min (0.01 mg/mL: p = 0.0004; 0.05 mg/mL: p = 0.0019) compared to TNF stimulation with particle activation.

An increased expression of *SOD2* was observed after 72 h + 45 min treatment with TNF + 0.01 mg/mL CoCr and TNF + 0.05 mg/mL CoCr compared to untreated control (all: p < 0.0001), 4 h + 45 min treatment (TNF + 0.01 mg/mL: p = 0.0086; TNF + 0.05 mg/mL: p = 0.0058) and particle exposure (all: p < 0.0001) (Fig. 5c). Examination of the mitochondrial membrane potential showed that 0.05 mg/mL CoCr treatment resulted in an increase in fluorescence signal at 72 h + 45 min, which was significant compared to 4 h + 45 min treatment (p = 0.0046). 72 h + 45 min treatment with 0.01 mg/mL CoCr caused a significant reduction compared to 4 h + 45 min (p = 0.0471). Treatment with TNF + 0.05 mg/mL CoCr at 72 h + 45 min was significantly higher compared to 4 h + 45 min (p = 0.0209) (Fig. 5d). Microscopic images showed strong and uniform staining of mitochondria in the TNF-pretreated osteoblasts, whereas the staining of the CoCr-treated cells was unevenly distributed and even sporadic around the nucleus (Fig. 5a).

**Fig. 6:**
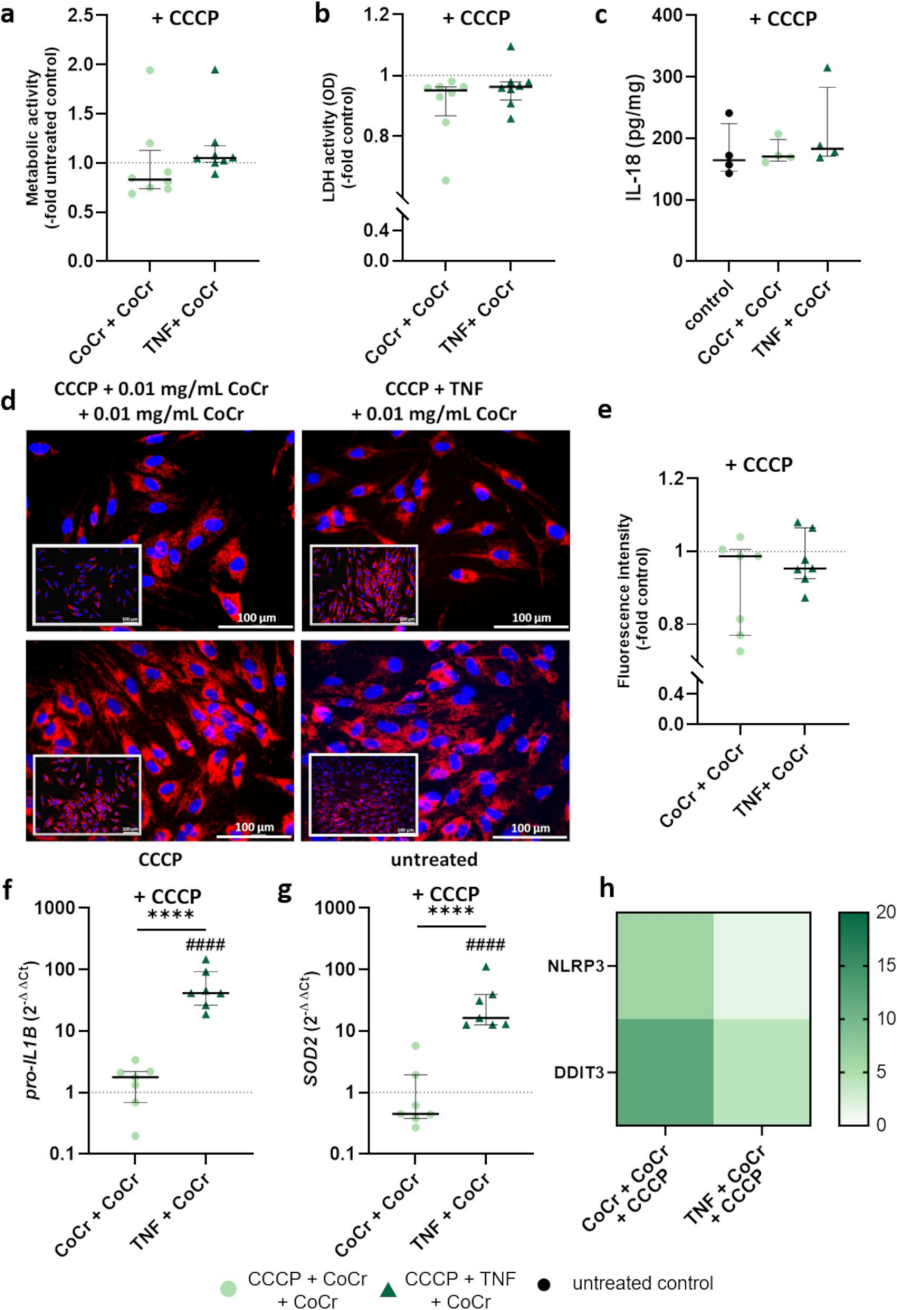
Determination of metabolic activity **a**, LDH activity **b**, IL-18 release **c**, and mitochondrial membrane potential **d**, **e** as well as gene expression analyses **f**–**h** after incubation of human osteoblasts (n ≥ 4) with CCCP and subsequent exposure to TNF or 0.01 mg/mL CoCr particles. After 72 h, the cells were again incubated with 0.01 mg/mL CoCr particles for 45 min. Data are presented as individual values with median and interquartile range **a**-**h** or in heat maps where the color coding corresponds to the mean. Normal distribution was tested using the Shapiro–Wilk test. Statistical significance was determined by unpaired t-test or Mann–Whitney test: *p < 0.05; **p < 0.01 (Significance between treatments); ^#^p < 0.05; ^##^p < 0.01 (Significance to corresponding control); Bar: 100 µm

To determine if there is a direct influence of mitophagy on the activation of the NLRP3 inflammasome after treatment with CoCr particles and TNF, osteoblasts were treated with the mitophagy agonist CCCP. No significant differences in metabolic activity (Fig. 6a), LDH activity (Fig. 6b), IL-18 release (Fig. 6c), or mitochondrial membrane potential (Fig. 6d-e) were observed between the treatments compared to the control. While CCCP + CoCr + CoCr treatment had no effect on the gene expression of *pro-IL1B* (Fig. 6f) and *SOD2* (Fig. 6g), significantly increased mRNA levels of *pro-IL1B* and *SOD2* were detected after TNF + CoCr treatment compared to control (*pro-IL1B*: p < 0.0001; *SOD2*: p < 0.0001) and CoCr + CoCr treatment (*pro-IL1B*: p < 0.0001; *SOD2*: p < 0.0001). The expression of *NLRP3* was unchanged in both treatments compared to the control. *DDIT3* mRNA levels were slightly increased after treatment with CCCP + CoCr + CoCr compared to the control (Fig. 6h).

## Discussion

A complex interplay of several intracellular signaling cascades induces the pathogenesis of aseptic loosening of joint endoprostheses. So far, the exact processes and responses that lead to aseptic implant loosening with periprosthetic osteolysis are not fully understood, but the initial focus is on local inflammation. This inflammation is triggered by pro-inflammatory mediators secreted after phagocytosis of wear debris. These include TNF and IL-1β. IL-1β secretion is regulated by the NLRP3 inflammasome. First, transcription of pro-IL-1β is induced by an NF-κB-activating priming signal, and then the preform is cleaved by caspase-1 into mature IL-1β and released. The production and release of IL-18 are also linked to the NLRP3 inflammasome. (Swanson et al. 2019) Preliminary work showed that metallic particles could affect the transcription of NLRP3-associated genes, but no activation of the NLRP3 inflammasome through the release of IL-1β could be detected (Sellin et al. 2024). The question arose whether the concentration of particles was too low or the single particle treatment was not sufficient to induce the NLRP3 inflammasome response. TNF has also been identified as a potent priming stimulus in human osteoblasts as an alternative to LPS priming. Therefore, the effects of double treatment of human osteoblasts with CoCr alone or in combination with TNF were initially investigated in this study.

Since primary human osteoblasts have only been treated with single CoCr particles at concentrations of 0.01 mg/mL and 0.05 mg/mL in previous studies (Sellin et al. 2024; Klinder et al. 2018; Jonitz-Heincke et al. 2018), the toxicity of a time-shifted multiple exposure to CoCr particles was first investigated. A concentration of 0.01 mg/mL CoCr particles is actually in the sub-toxic range (Klinder et al. 2018). However, no significant difference in cell viability was observed between the concentrations. Interestingly, after 4 h + 45 min, there was a tendency for the lower particle concentration to have a more toxic effect on osteoblasts. This effect was also observed after pretreatment with TNF and activation with 0.01 mg/mL or 0.05 mg/mL. This observation was seen in previous studies and may be related to particle size. The size and composition of particles play a significant role in their uptake by osteoblasts and subsequent effects. Wear particles released from joint endoprosthesis can vary in size. The average size of CoCr particles isolated from periprosthetic tissue is in the nanometer range (Athanasou 2016; Doorn et al. 1998). Although it has been demonstrated that CoCr particles ranging from 0.1 µm stimulate the NLRP3 inflammasome response in MG63 osteoblasts (Brunken et al. 2023), future studies should examine the impact of smaller particle sizes within a clinically relevant range. However, studies showed that wear particles within a size range from nanometers to micrometers can activate immune and bone cells (Stratton-Powell et al. 2022; Ormsby et al. 2019). The particles used in this study had a mean size of 0.5 µm. Osteoblasts are able to ingest smaller particles (less than 20 µm) more quickly and induce a stronger inflammatory response (Yang et al. 2019; Xu et al. 2018). The higher the particle concentration, the more likely it is to agglomerate, making it more difficult for the particles to be taken up by the cells (Klinder et al. 2018). The heterogeneity of the agglomerates could also lead to distinct cellular reactions compared to the single particles (Caicedo et al. 2013), as besides the size, the shape also influences the NLRP3 inflammasome. Larger and irregularly shaped particles increase the intracellular lysosomal damage, which activates the inflammasome. As the results of the particle stimulations do not have high deviations, it is assumed that the agglomerates are homogeneous. However, over time, corrosion and ion release may occur, affecting cell viability (Jonitz-Heincke et al. 2019). Further studies should measure the ion concentration in the cell culture supernatants and determine the influence on viability. It can be concluded that the toxicity of these CoCr particles and the concentrations used are strongly dependent on the exposure time. This hypothesis was further confirmed by the fact that the second treatment of the cells with particles for 45 min had no relevant influence on the viability of the cells. Treatment with TNF also led to a slight decrease in viability and an increase in the number of dead cells over time, but this was not as strong as after particle treatment.

Priming of human osteoblasts with 0.01 mg/mL CoCr particles induced the mRNA levels of the inflammasome-associated genes *TLR2*, *TLR4*, *NLRP3,* and *pro-IL1B* with increasing exposure time (Sellin et al. 2024). In this study, an increase in gene expression levels was also observed, although no influence of the activation signal on the transcription levels of the various genes could be detected. On the one hand, this confirms the existing concept that the activation signal does not influence the transcription of the genes. On the other hand, the second stimulation time might be too short. Again, a time-dependent influence was shown, although in contrast to the prevailing model, in which immune cells are primed for four hours and then activated, significantly more extended periods were required before an influence of the particles on gene expression could be detected (Jämsen et al. 2020). In comparison, treatment of osteoblasts with TNF resulted in increased mRNA levels of *pro-IL1B* after only four hours. This increase in expression is due to the influence of TNF.

Despite increased levels of gene expression and re-addition of particles, no release of mature IL-1β was detected. Another cytokine cleaved by the NLRP3 inflammasome is IL-18. Osteoblasts are able to secrete IL-18, and they can also respond to IL-18 via IL-18-Receptor (Udagawa et al. 1997; Cornish et al. 2003). IL-18 plays a pleiotropic role in bone metabolism. Depending on the environment, it can have a pro-osteogenic effect by inducing osteoblast proliferation and inhibiting osteoclast formation. When IL-18 interacts with other cytokines, it can have a pro-osteoclastogenic impact by influencing the RANKL/osteoprotegerin ratio in favor of RANKL, thereby inducing osteoclastogenesis (Mansoori et al. 2016). No change in pro-IL-18 gene expression could be detected, but the release of mature IL-18 is not dependent on prior activation of transcription, as cells constitutively express pro-IL-18 mRNA (Puren et al. 1999; Jämsen et al. 2020). Another reason for the difficulty in detecting pro-IL18 mRNA levels is the reported short half-life. In macrophages, a half-life of 4.97 min has been reported, depending on the microenvironment (Satwani et al. 2005). Gene expression remained unchanged, yet IL-18 release was detected, indicating a time-dependent influence of the second stimulus. The release of IL-18 showed that the NLRP3 inflammasome was activated after particle exposure. Renewed addition of particles, regardless of concentration, triggered a significant IL-18 release after 4 h and 45 min. Additionally, an increased baseline release was observed after 72 h, independent of the activation signal. This effect is likely due to the long-term treatment of the osteoblasts, making the cells less sensitive to renewed treatments. These findings indicate that after short-term priming, the activation of the NLRP3 inflammasome depends on a new, externally supplied activation signal; in contrast, more extended exposure to CoCr particles induces different internal processes leading to cell death and secretion of inflammatory mediators, cellular components, and ingested particles, which can trigger the activation of inflammasome without a new activation stimulus (Swanson et al. 2019; Akbal et al. 2022). Furthermore, the amount of dead cells was very high after 72 h of particle exposure compared to 4 h. This indicates that the dying cells released the protein over time, and only the remaining living cells react to the second stimulus with the secretion of IL-18. However, it's crucial to note that the half-life of the IL-18 protein in the circulation is only a few Hours, so the endpoint measurement after 72 h ± 45 min only provides information about the current release rate. The increased release of IL-18 in the untreated control after 72 h ± 45 min is attributable to the baseline production of IL-18 as a mediator of osteoblast differentiation (Mansoori et al. 2016). The differentiation of osteoblasts can also be influenced by the NLRP3 inflammasome. Detzen et al. found that the expression of NLRP3 is necessary for cell differentiation, while overexpression during inflammation leads to disruption of the process (Detzen et al. 2021). In this study, the particle treatment alone led to reduced expression rates of the differentiation markers runt related transcription factor 2 (*RUNX2*), collagen type 1 (*COL1A1*), and alkaline phosphatase (*ALPL*) (Supplement Fig. S1). While it has been reported that TNF slows down osteogenic differentiation (Xia et al. 2023) by activating the NLRP3 inflammasome and inducing pyroptosis, our results showed that treatment of osteoblasts with TNF and particles for 72 h + 45 min led to the induction of *RUNX2* and *COL1A1*, while *ALPL* was reduced. Since it was shown that inhibiting the NLRP3 activity led to an increase in differentiation and bone formation markers (Xu et al. 2024), the effect of inhibiting the particle-induced NLRP3 activation should be investigated in detail in future studies.

LDH activity directly correlates with IL-18 release after 4 h + 45 min, indicating the induction of pyroptosis. Light microscopic images (Supplement Fig. S2) showed that after all treatments, the osteoblasts were rounded and appeared swollen. Since pyroptosis induces cell swelling and membrane rupture, these morphologic changes indicate pyroptosis. The cell morphology of the osteoblasts changed significantly after the treatments, especially after the treatment with the highest particle concentration. The cells had no clear structure, which indicated the toxicity of the high CoCr particle concentration. The morphological changes of the osteoblasts have to be analyzed with electron microscopy in future studies.

Furthermore, an influence of actin remodeling after particle treatment on the gene expression profiles of proinflammatory cytokines was found in osteoprogenitor cells (Lee et al. 2011). In contrast to our study, these osteoprogenitor cells exposed to titanium particles did not show increased expression of TNF or IL-1β. However, activation of the ERK signaling pathway via CCAAT/enhancer binding protein β (CEBP-β) was detected, leading to increased IL-6 gene expression. The reaction of cells to wear particles is strongly dependent on their composition (Lohmann et al. 2000). Brunken et al. showed that titanium particles do not activate the NLRP3 inflammasome. Still, CoCr particles influence it (Caicedo et al. 2013). Therefore, the results of Lee et al. could be due to the lack of influence of titanium on the NLRP3 inflammasome (Lee et al. 2011). CoCr particles induced the increase of inflammasome-associated genes independent of the NF-κB signaling pathway. The impact of actin remodeling on gene expression profiles after particle exposure provides a signaling pathway that is primarily independent of NF-κB. Further studies must investigate the influence of particle-mediated actin remodeling on the inflammatory response in more detail using the inhibition of actin polymerization.

In osteoblasts, the NLRP3 inflammasome contributes to titanium particle-induced osteolysis by inducing inflammation and apoptosis (Yu et al. 2023). Therefore, it can be assumed that exposure to CoCr particles leads to a combination of different cell deaths. Even though NLRP3 activation is mainly linked to pyroptosis, the functional expression of NLRP3 is also known to directly influence apoptosis by reducing NF-κB activity (McCall et al. 2008). NF-κB plays an important role in the inhibition of TNF-initiated apoptosis (You et al. 2001). In our previous study, we were able to demonstrate activation of NF-κB with simultaneous oscillating NLRP3 gene expression following TNF stimulation, and human osteoblasts exhibited good cell survival. In contrast, exposure to CoCr particles did not lead to NF-κB activation, but to an increase in NLRP3 transcripts over the stimulation period (Sellin et al. 2024). Most recently, the study by Yu et al. (2023) also showed that titanium particle-induced ROS, combined with mitochondrial dysfunction and increased NLRP3 expression, induces inflammatory cytokines and apoptosis in osteoblasts. We also proved mitochondrial dysfunction with the production of ROS (Sellin et al. 2024) and the reduction of mitochondrial membrane potential after CoCr exposure, which might contribute to apoptotic processes in our study. Future studies will examine the processes leading to cell death triggered by abrasion particles in greater detail.

Wear particles and the released inflammatory mediators induce and amplify ER stress by increasing protein synthesis and folding, which exceeds the ability of the ER to remove misfolded proteins. This results in an accumulation of unfolded and misfolded proteins and increased production of DDIT3, which can regulate the apoptosis of osteoblasts during ER stress. Recently, the link between ER stress and inflammasome signaling pathways has been established (Lebeaupin et al. 2015; Lerner et al. 2012; Oslowski et al. 2012). Dong et al. (Dong et al. 2024) found that the increased production of DDIT3 is associated with the activation of the NLRP3 inflammasome. Increased gene expression of DDIT3 was detected in the particle-treated osteoblasts of this study. An increased DDIT3 level may indicate an inhibition of mitophagy, which leads to increased ROS levels so that the NLRP3 inflammasome can be activated (Dong et al. 2024). Mitophagy plays a protective role against inflammation by eliminating damaged mitochondria.

Preliminary work has shown that CoCr particles induced ROS in human osteoblasts (Sellin et al. 2024). The evaluation of the mitochondrial membrane potential revealed the initial effects of the particles and TNF on the mitochondria. During depolarization of the mitochondrial membrane potential, mitophagy is activated in the affected mitochondria via the PINK1/Parkin signaling pathway, and the mitochondria are degraded (Nguyen et al. 2016). While osteoblasts in the control group had homogeneously distributed, intact mitochondria, the particle treatment resulted in morphological changes and loss of mitochondria. There was also condensation in the region of the cell nucleus. Damage to mitochondria reduces cell viability and mineralization capacity and can induce cell death at high levels (Gao et al. 2018; Ho et al. 2005; Chae et al. 1997). The loss of mitochondrial membrane potential once again illustrates the cytotoxic effect of CoCr particles. Wang et al. (2020) demonstrated that titanium particles could penetrate the mitochondria, inducing mitochondrial dysfunction (Wang et al. 2020). Future experiments should investigate whether these CoCr particles can also penetrate the mitochondria and cause direct damage. In further studies, the influence of metallic particles and TNF on the expression of mitophagy-associated proteins such as PINK1, LC3BI, LC3BII, and p62 should be investigated.

The regulatory mechanisms of the antioxidative systems against increased levels of ROS influence bone formation (Arai et al. 2007; Gao et al. 2018). The manganese superoxide dismutase (SOD2) is localized in the mitochondria and protects the cells from oxidative damage by converting superoxide radicals to oxygen and hydrogen peroxide (Gao et al. 2018). When looking at *SOD2*, an increase in expression was observed after TNF pretreatment and subsequent CoCr activation, which can be attributed to the effect of TNF, as no change in expression could be detected in the CoCr + CoCr-treated samples. Gao et al. showed that overexpression of *SOD2* was induced to maintain a low mitochondrial ROS concentration (Gao et al. 2018). Low SOD2 levels lead to an increase in ROS, inducing mitophagy (Tang et al. 2024). The results of this study suggest that activation of the inflammasome by TNF may be more reversible as cells attempt to restore cell function by increasing SOD2. In contrast, exposure to particles leads to irreversible cell destruction.

Removal of damaged mitochondria and high levels of ROS could be a key factor in reversing the particle-induced inflammatory response. For this reason, mitophagy was induced by the addition of carbonyl cyanide m-chlorophenyl hydrazine (CCCP), a mitophagy agonist, in the TNF- and particle-exposed cells. CCCP is a protonophore that increases proton conductance in the membrane, leading to mitochondrial depolarization. This blocks the import of PINK1 so that it accumulates in the outer mitochondrial membrane, and the degradation of the damaged mitochondria is induced by the activation of mitophagy (Yoshii et al. 2011; Padman et al. 2013; Kasianowicz et al. 1984). In the study by Dong et al., the inhibition of mitophagy by DDIT3 was reversed in the presence of CCCP (Dong et al. 2024). In our present study, the addition of the agonist was able to inhibit the effects of the particles. Improved metabolic activity and reduced LDH release were detected compared to treatments without CCCP.

Furthermore, the release of IL-18 was reduced so that the inflammatory reaction was diminished. The addition of CCCP also increased the mitochondrial membrane potential during particle exposure. The induction of mitophagy did not seem to affect the gene expression of *DDIT3* after particle treatment, and increased mRNA levels were still detected. This result indicates that CCCP has no effect on the transcriptional regulation of *DDIT3* and should be verified by protein expression analysis. Strikingly, osteoblasts treated twice with CoCr particles show gene expression of *pro-IL1B* at the level of the control. In contrast, the expression was reduced upon TNF exposure compared to treatment without CCCP but remains elevated. This result suggests that the transcriptional enhancement by CoCr particles may be influenced by a mitophagy-dependent signaling pathway, whereas the gene expression enhancement by TNF is only partially influenced by it.

This study has some limitations. While this study records changes in gene expression and the presence of the protein qualitatively, it was not possible to quantify the protein synthesis rate of associated proteins. It is well established that mRNA levels do not always correlate directly with corresponding protein levels, since post-transcriptional regulation, translation efficiency, and protein degradation can all play a significant role. Since proteins are the actual functional carriers within cells, it is not possible to draw direct conclusions about functional consequences from gene expression rates alone. Therefore, the exact biological effect of the observed gene regulation remains unclear. Despite the absence of protein analysis, investigating gene expression provides essential initial insights into the molecular changes under the studied conditions. Changes in gene expression can provide an early indication of regulatory processes that can be explored in greater depth through further analysis. This study provides a solid foundation for subsequent functional studies at the protein level.

## Conclusion

The data of our present study show that brief priming with CoCr particles or TNF, followed by CoCr exposure as an activation signal, can activate the NLRP3 inflammasome in human osteoblasts. Two conclusions can be drawn from this result: 1) Priming of human osteoblasts with TNF or CoCr followed by activation with particles induces a similar strong inflammatory response via the NLRP3 inflammasome. 2) Activation of the NLRP3 inflammasome after short-term treatment is dependent on a new, externally supplied activation signal. In contrast, long-term treatment of the cells induces inflammatory processes that can activate the NLRP3 inflammasome independently of an external signal. At the same time, our results show that the particles induce the ER stress marker *DDIT3* and reduce the degradation of damaged mitochondria. As mitophagy induction leads to improved cell survival and reduced IL-18 secretion, an influence of the particles on a mitophagy-dependent signaling cascade can be assumed. This observation should be further analyzed to elucidate the intracellular particle-induced signaling pathways.

## Supplementary Information

Below is the link to the electronic supplementary material.

Supplementary file1 (DOCX 3133 KB)

## Data Availability

The datasets generated and/or analyzed during the current study are available from the corresponding author upon reasonable request.

## Declarations

### Clinical trial number

Clinical trial number: not applicable.
