# Supplementary material for: Repeated exposure to CoCr28Mo6 particles leads to activation of NLRP3 inflammasome signaling in human osteoblasts
Source: Cell Biol Toxicol. 2025 Sep 23;41(1):131. doi: 10.1007/s10565-025-10087-7 (PMC12457469; doi:10.1007/s10565-025-10087-7)
Supplement: Supplementary file 1 — Supplementary file1 (DOCX 3133 KB) [file 10565_2025_10087_MOESM1_ESM.docx]

**Supplementary Information**

Repeated exposure to CoCr28Mo6 particles leads to activation of NLRP3 inflammasome signaling in human osteoblasts

Marie-Luise Sellin ^1^, Luca Marit Köntopp ^1^, Rainer Bader ^1^, and Anika Jonitz-Heincke ^1^

^1^ Department of Orthopaedics, Biomechanics and Implant Technology Research Laboratory,

Rostock University Medical Center, 18057 Rostock, Germany

marie-luise.sellin@med.uni-rostock.de (ORCID: 0009-0008-1231-7132)

luca.koentopp@uni-rostock.de

rainer.bader@med.uni-rostock.de

anika.jonitz-heincke@med.uni-rostock.de (ORCID: 0000-0001-9318-4264)

**Correspondence to:**

Marie-Luise Sellin

Department of Orthopaedics, Biomechanics and Implant Technology Research Laboratory

Rostock University Medical Center

Doberaner Straße 142

18057 Rostock, Germany

Telephone: +49 381 494-9306

Fax: +49 381 494-9308

**Figure S1:**


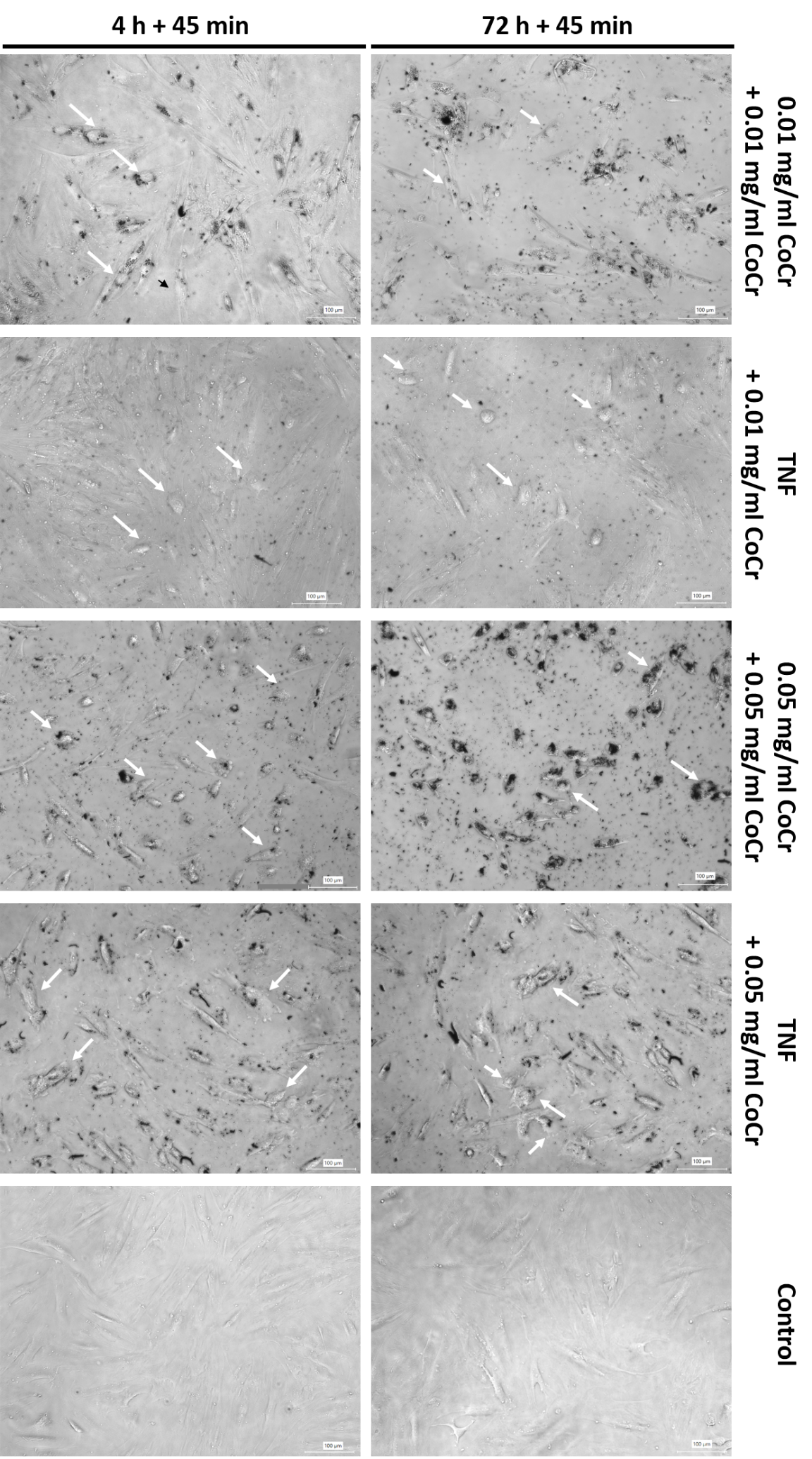


↑ Fig. S1: Light microscopic images of human osteoblasts after priming with cobalt-chromium-molybdenum (CoCr) particles or tumor necrosis factor (TNF) for 4 h (left side) or 72 h (right side) and subsequent activation with CoCr particles for 45 min. The white arrows indicate swollen cells. Images were taken with a Keyence BZ-X810 microscope with a 20x objective. Bar: 100 µm

**Figure S2:**

**Method: ALP staining**

ALP staining was performed using Liquid Permanent Red (Agilent Technologies, Inc., Santa Clara, California, USA). The osteoblasts were washed twice with PBS after treatment, then fixed with 100 % ethanol for 10 minutes. Following fixation, the cells were washed with TRIS buffer (50 nM, pH=8.0) and incubated with the staining solution in the dark for 30 minutes. The staining solution was prepared according to the manufacturer's instructions. After the incubation period, the solution was removed, and the cells were washed with ultrapure water. The fluorescence signal was measured using a plate reader (Tecan Group AG, Männedorf, Switzerland) at a wavelength of 630 nm, and the samples were recorded at 10x objective using a Keyence BZ-X810 microscope (Keyence Germany GmbH, Neu-Isenburg, Germany).

Table S1: cDNA target sequences for semi-quantitative real-time PCR (Primer pairs were purchased from Merck KGaA (Darmstadt, Germany)


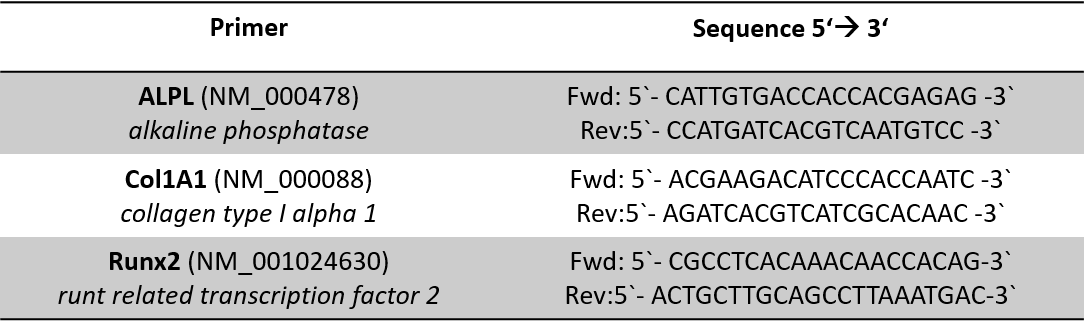


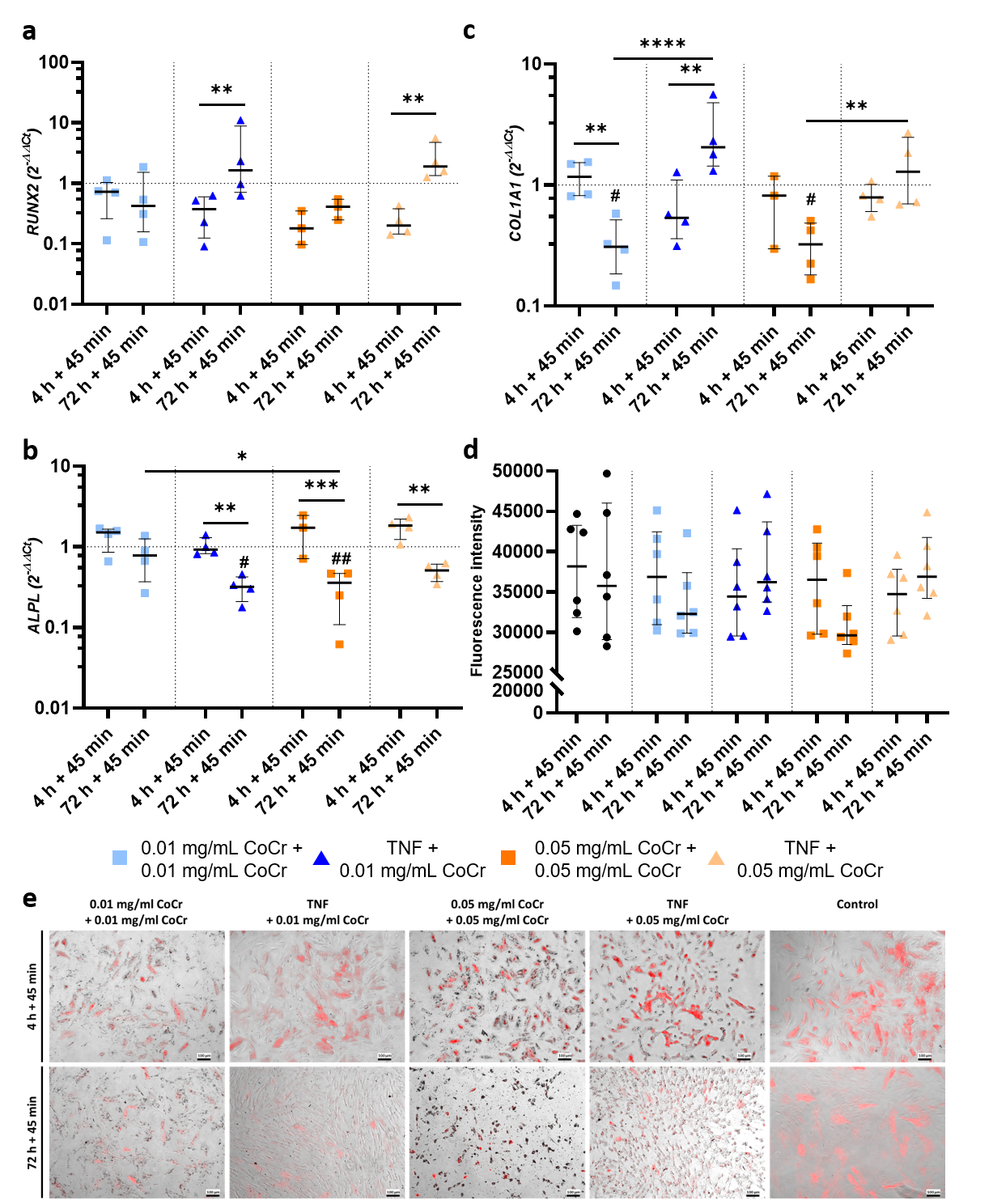


↑ Fig. S2: Determination of gene expression of RUNX2 (a), COL1A1 (b), and ALPL (c) as well as ALP activity (d) after priming of human osteoblasts (n=4) with CoCr particles or TNF and subsequent activation with CoCr particles. Light microscopic images of human osteoblasts after priming with CoCr particles or TNF for 4 h (left side) or 72 h (right side) and subsequent activation with CoCr particles for 45 min. Images were taken with a Keyence BZ-X810 microscope with a 10x objective. Gene expression data were calculated using the 2^-ΔΔCt^-method (-fold untreated control, dashed line). Statistical significance was determined by two-way ANOVA followed by Bonferroni post hoc test: ****p<0.0001, ***p<0.001, **p<0.01, *p<0.05; ^##^p<0.01, ^#^p<0.05 (Significance to untreated control). Bar: 100 µm
